# SARS-CoV-2 Surface Contamination and Viable Virus in High-Ventilation Single Rooms: Implications for Fomite Transmission

**DOI:** 10.14789/ejmj.JMJ25-0037-OA

**Published:** 2026-03-19

**Authors:** ATSUKO HAYASE, MASAYUKI OGATA, MAKOTO HIKI, YASUHIRO OKADA, SHUN KUBO, MARI SATO, TOSHIO NAITO, YOKO TABE, KENJI MANABE, TAKUYA MORI, NORIYASU OTA, SATOSHI HORI

**Affiliations:** 1 Biological Science Research Laboratory, Kao Corporation, Tochigi, Japan; 1 Biological Science Research Laboratory, Kao Corporation, Tochigi, Japan; 2 Department of Architecture, Faculty of Urban Environmental Sciences, Tokyo Metropolitan University, Tokyo, Japan; 2 Department of Architecture, Faculty of Urban Environmental Sciences, Tokyo Metropolitan University, Tokyo, Japan; 3 Department of Emergency and Disaster Medicine, Juntendo University Faculty of Medicine, Tokyo, Japan; 3 Department of Emergency and Disaster Medicine, Juntendo University Faculty of Medicine, Tokyo, Japan; 4 Personal Health Care Products Research, Kao Corporation, Tokyo, Japan; 4 Personal Health Care Products Research, Kao Corporation, Tokyo, Japan; 5 Department of General Medicine, Juntendo University Faculty of Medicine, Tokyo, Japan; 5 Department of General Medicine, Juntendo University Faculty of Medicine, Tokyo, Japan; 6 Department of Clinical Laboratory Medicine, Juntendo University Graduate School of Medicine, Tokyo, Japan; 6 Department of Clinical Laboratory Medicine, Juntendo University Graduate School of Medicine, Tokyo, Japan; 7 Bioresource Research Center, Juntendo University Graduate School of Medicine, Tokyo, Japan; 7 Bioresource Research Center, Juntendo University Graduate School of Medicine, Tokyo, Japan; 8 Department of Infection Control Science, Juntendo University Graduate School of Medicine, Tokyo, Japan; 8 Department of Infection Control Science, Juntendo University Graduate School of Medicine, Tokyo, Japan

**Keywords:** SARS-CoV-2, COVID-19, fomite transmission, environmental surface contamination, infectious dose

## Abstract

**Objectives:**

To evaluate Severe Acute Respiratory Syndrome Coronavirus 2 (SARS-CoV-2) contamination in the rooms of Coronavirus disease 2019 (COVID-19) patients by integrating RNA quantification, viral culture, and post-culture cycle threshold (Ct)-based estimation of infectious titer, and to reassess fomite transmission risk relative to the human infectious dose.

**Methods:**

In nine high-ventilation, single-occupancy rooms, adhesive 36 cm^2^ samplers (stickers for hard surfaces; cloth patches for textiles) were placed on 14 environmental surfaces and on the upper back of night-shift nurses’ scrubs for the first 24 hours after admission. N-gene copies were quantified by quantitative reverse transcription PCR (qRT-PCR); infectivity was assessed on VeroE6/TMPRSS2 cells. For culture-positive samples, culture-supernatant Ct values were converted to estimated infectious titers using a published regression. Distance, material, and Severity classification factors were analyzed in prespecified univariable models.

**Results:**

Of 134 specimens, 66 (49.3%) were RNA-positive, with a maximum of 8.48 log_10_ copies per sample, highest values on high-touch surfaces close to patients. Infectious virus was isolated from 4/134 specimens (3.0%). Estimated infectious titers were approximately 5.7 × 10^2^-2.6 × 10^3^ TCID_50_-eq per sample. Both RNA positivity and viral RNA copy numbers declined significantly with increasing distance from the patient’s mouth.

**Conclusions:**

In high-ventilation rooms, environmental surface contamination was common and viable virus was occasionally present near patients. Estimated titers on some high-touch surfaces exceeded the reported human infectious dose, implying that fomite transmission may remain plausible under specific conditions. Prioritized cleaning/disinfection of high-touch surfaces near patients, along with consistent hand hygiene, are warranted, alongside ventilation/filtration measures.

## Introduction

To curb the spread of coronavirus disease 2019 (COVID-19), it is essential to understand the transmission modes of Severe Acute Respiratory Syndrome Coronavirus 2 (SARS-CoV-2) and to adopt appropriate infection control measures. Respiratory viruses including SARS-CoV-2 are transmitted primarily via direct deposition onto mucosal surfaces, enabling person-to-person horizontal transmission; however, under special circumstances such as “3Cs” conditions (closed spaces, crowded places, close-contact settings), infection can occur via inhalation of infectious respiratory particles^1)^. In addition, although less frequently, fomite transmission has been reported; after contact with pathogen-contaminated environmental surfaces or objects, infectious particles are transferred via the hands to the mucous membranes of the mouth, nose, or eyes^1-5)^.

Multiple studies have documented the detection of SARS-CoV-2 RNA on environmental surfaces particularly in healthcare settings^3)^. For example, an environmental survey of inpatient rooms in Japan reported an RNA qualitative positivity rate of 12.4% (105/849 samples), while infectious virus was isolated from 0.47% samples (4/849 samples; 7-290 PFU/mL)^6)^. In a Canadian hospital, the positivity rate by quantitative RNA testing was 33% (151/459 samples), and infectious virus was isolated in 2-19% of samples (5-2000 PFU/mL)^7)^.

There is also a growing body of evidence regarding the minimal infectious dose in humans. In a human challenge study of healthy young adults, intranasal inoculation with 10 50% Tissue Culture Infection Dose (TCID_50_) (55 FFU) led to infection in approximately half of participants, suggesting that even small exposures can result in infection under certain conditions^8)^. This finding implies that, although infrequent, fomite transmission can occur.

In this study, during the early pandemic period dominated by the SARS-CoV-2 Delta variant, we conducted 24-hour deposition sampling in the rooms of patients with COVID-19, performing RNA quantification by quantitative reverse transcription PCR (qRT-PCR) and culture-based assessment of infectivity. Furthermore, we converted post-culture cycle threshold (Ct) values to estimated infectious titers (TCID_50_-eq/mL) using a published approximation method and, by comparing these estimates with current evidence on the minimal infectious dose in humans, aimed to re-evaluate, quantitatively, the risk of fomite transmission from the standpoint of whether detected infectious virus could reach the infection threshold.

## Materials and Methods

### Participant recruitment and study participation

We enrolled adults (≥ 20 years) with confirmed SARS-CoV-2 infection who were within five days of symptom onset and had mild-to-moderate severity classification. Patients were admitted to Juntendo University Hospital between April and May 2021. Severity of classification was based on the Japanese Clinical Practice Guide for COVID-19 (version 4.2) comprising four categories: Minor (no pneumonia or hypoxemia), Moderate I (pneumonia without respiratory failure), Moderate II (respiratory failure requiring oxygen therapy; SpO_2_ ≤ 93%), and Severe (ICU admission and/or mechanical ventilation). Only Minor, Moderate I, and Moderate II cases were included in this study. After written informed consent, participants were admitted to single-occupancy isolation rooms with a private toilet and shower. For nursing-scrub sampling, nurse participants were ward nurses caring for patients with COVID-19 who consented to participate for a full shift.

### Room settings

Each isolation room had a volume of 38 m^3^ with an outdoor-air ventilation rate of 456 m^3^/h (equivalent to 12 air changes per hour, ACH). In addition, a high-efficiency particulate air (HEPA)-filter air purifier with an equivalent airflow of 583 m^3^/h was operated in the room. The set room temperature and relative humidity were 25°C and 50%, respectively.

### SARS-CoV-2 environmental sampling

**Figure 1:**
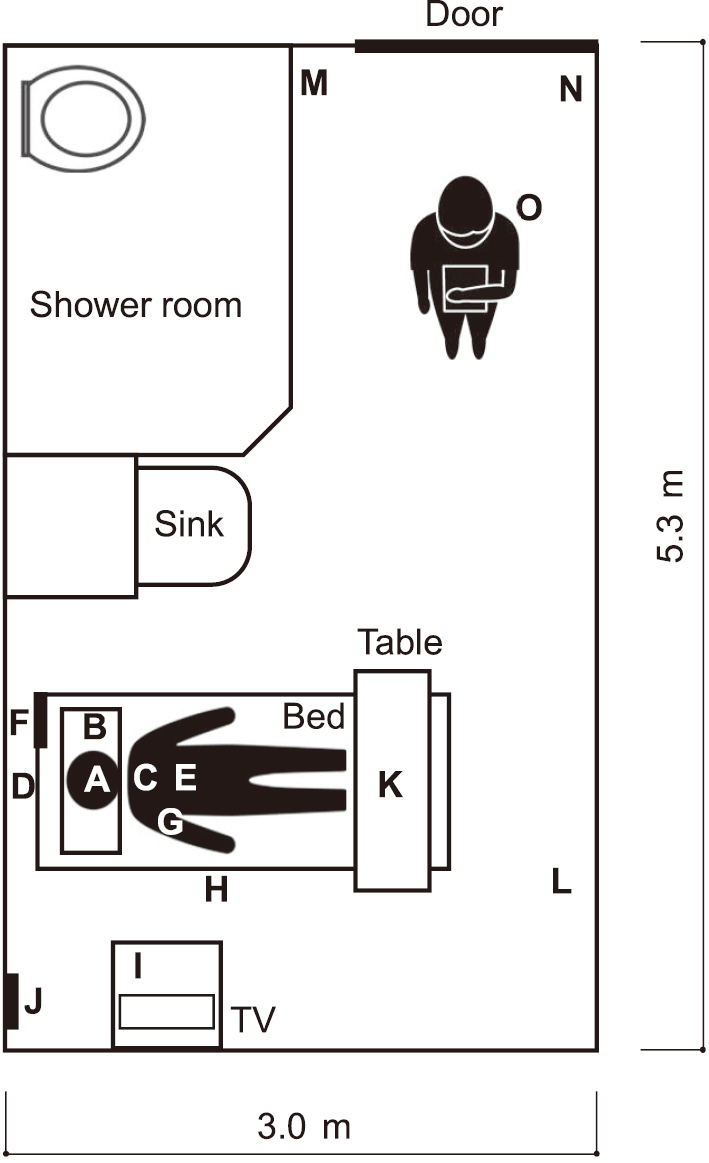
Sampling site Fourteen sites were specified in the patient room (A-N), and one site was located on the upper back of the night-shift nurse’s scrub (O). A, surgical mask (a 6 cm × 6 cm piece excised from the nose and mouth contact portion); B, pillowcase (four sites by the head); C, pajamas (two bilateral sites from the pectoral region and cuff); D, bed railing (overhead); E, smartphone screen; F, nurse call button; G, TV remote controller; H, bed railing (side, bilateral); I, TV stand; J, light switch; K, table (two bilateral sites); L, floor; M, doorknob (shower room); N, doorknob (hospital room, inside); O, upper back of the nurse’s scrub. For subjects with multiple sampling sites, a test was considered positive if viral RNA was detected in any of the samples.

Sampling locations comprised 14 sites within each room plus the upper back panel of night-shift nurses’ scrubs, totaling 15 sites (Figure 1). Instead of manual swabbing, we used an adhesive sampling method. On hard surfaces, 6 × 6 cm (36 cm^2^) polypropylene stickers (3M) were applied; on textiles (pillowcases, pajamas), cloth patches made of the same material were attached. All samplers were placed immediately prior to patient entry to the room and retrieved 24 hours later. For surgical masks worn by patients, after 24 hours of use, a 6 × 6 cm piece was excised from the central upper area that directly contacted the nose and mouth. During the first 24 hours after admission (the environmental sampling period), oxygen saturation on room air (SpO_2_) was monitored; the maximum and minimum values were recorded. Samplers on nurses’ scrub tops were affixed at the beginning of the night shift; accordingly, scrub sampling reflects the night shift only. The upper back was selected a priori based on infection-prevention considerations and feedback from ward nurses. At the time of the study (early 2021), the transmission characteristics of this emerging infection were not fully clarified, and the available PPE configuration did not fully cover the upper back. This area was also considered potentially vulnerable to secondary contamination during gown doffing. In response to nurses’ concerns regarding possible viral deposition at this partially exposed site, and to minimize disruption and self-contact compared with a frontal placement, we selected the upper back as the sampling location.

### Virus recovery and concentration

Recovered samplers were placed into 50 mL tubes containing 20 mL of 0.05% Tween 80 solution and eluted using vortex mixing. Fifteen milliliters of the eluate were transferred to an ultrafiltration tube (Amicon Ultra-15, 30K; Merck) and centrifuged at 2,000 × g for 30 min at 25°C. The supernatant was collected and concentrated in 0.05% Tween 80 solution to 300 μL. Of this concentrate, 140 μL was used for RNA extraction; the remainder was supplemented with 1 mL of Universal Viral Transport Medium (Becton Dickinson) for culture.

To contextualize recovery, our concentration workflow followed Ahmed et al., who reported a mean recovery of approximately 56.0% for viruses concentrated from solution using a similar ultrafiltration protocol^9)^. Prior to the study, we conducted a bench- top recovery assessment in which influenza virus was spiked onto adhesive samplers (polypropylene sticker and cloth patch) and processed with the identical elution (20 mL, 0.05% Tween 80) and ultrafiltration steps. The overall recovery, calculated as post-concentration copies divided by spiked copies, was 46% for sticker coupons and 31% for cloth coupons. In our workflow, 15 mL of the 20 mL eluate (75%) entered underwent ultrafiltration and was concentrated to 300 µL; of which 140 µL (46.7%) of the concentrate was used for RNA extraction and 160 µL (53.3%)—supplemented with 1 mL of transport medium—was used for culture.

### RNA extraction and qRT-PCR

RNA was extracted using the QIAamp Viral RNA Mini Kit (QIAGEN) and eluted in 60 μL in accordance with the manufacturer’s instructions. qRT- PCR was performed with the QuantiTect Probe RT-PCR Kit (QIAGEN) on an Mx3000P instrument (Agilent) targeting the SARS-CoV-2 N gene. Primers and probe followed the NIID protocol^10)^. Reverse transcription was carried out at 50°C for 30 min, followed by initial denaturation at 95°C for 15 min, then 50 cycles of 95°C for 15 s and 60°C for 1 min. A standard curve generated using synthetic RNA (Nihon Gene Research Laboratories, Inc.) was used to quantify copy numbers. Each specimen was tested in duplicate (n = 2), and mean values were used.

### Isolation of infectious virus

Isolation of infectious virus was outsourced to an external facility operating under BSL-3 conditions and conducted in 2021. VeroE6/TMPRSS2 cells (JCRB1819) were grown as monolayers in six-well plates using low-glucose Dulbecco's Modified Eagle Medium (DMEM) supplemented with 5% heat-inactivated FBS and 1 mg/mL G418. Culture samples were centrifuged at 3,600 × g for 5 min at 4°C and 300 μL of supernatant were inoculated into three wells and allowed to adsorb for 1.5 h. Cells were then washed with DMEM, overlaid with 3 mL/well of DMEM containing 1 mg/mL G418, and incubated at 37°C with 5% CO_2_ for 2-6 days. Cytopathic effects (CPE) were monitored daily by phase-contrast microscopy. For wells showing CPE, supernatants were collected and SARS-CoV-2 was confirmed by qRT-PCR using the SARS-CoV-2 Detection Kit N1 set (TOYOBO) in accordance with the recommended protocol: 6 μL of culture medium was mixed with 3 μL of pretreatment solution and heated at 95°C for 5 min, followed by addition of 40 μL of RT- PCR reaction mixture; reverse transcription and pre- denaturation were performed on a Thermal Cycler Dice Real Time System III (Takara), and PCR was run for 45 cycles.

### Estimation of infectious titer from culture-supernatant Ct values

Ct values from culture supernatants were converted to estimated infectious titers using the regression reported by Brandolini et al. (2021): log_10_ (TCID_50_/mL) = −0.3152 × Ct + 9.988^11)^. Results are expressed as TCID_50_ equivalents (TCID_50_-eq). This metric is a semi-quantitative indicator of the amount of virus amplified during culture and does not directly represent the infectious titer in the original specimen.

### Statistics

The primary outcomes were viral detection in environmental samples, RNA load (log_10_[copies]), and culture positivity. Viral detection and culture positivity were analyzed using logistic regression, whereas RNA load was analyzed using linear regression. Distance from the patient’s mouth was modeled as a continuous variable, and odds ratios and regression coefficients were estimated. Surface materials were classified as nonporous or textile, and comparisons between material types were conducted. Sensitivity analyses included: (i) models incorporating both material type and distance, and (ii) Firth’s penalized logistic regression to address potential small-sample bias in culture positivity. In a separate analysis, prespecified patient-related factors were evaluated for associations with RNA detection, RNA load, and culture positivity using univariable regression models. Effect sizes were reported with 95% confidence intervals, and a two-sided significance level of α = 0.05 was applied. All analyses were performed using R (version 4.5.2).

### Ethical approval

This study was approved by the Institutional Review Board of Juntendo University Hospital and the Ethics Committee of Kao Corporation (approval Nos. 20-334 and T324-201115). Written informed consent was obtained from all participants.

## Results

### Participant characteristics

Nine hospitalized patients with mild-to-moderate COVID-19 who met the inclusion criteria were enrolled (six male, three female). The median age was 64 years (range, 21-77 years) (Table 1). All eligible patients who provided consent were included; there were no exclusions or refusals.

**Table 1:** Information on study participants

Subject No.		#1	#2	#3	#4	#5	#6	#7	#8	#9
Patient condition on admission										
Age		69	64	29	21	77	65	44	72	37
Sex		M	F	M	M	F	M	M	F	M
Severity		Moderate II	Moderate I	Minor	Minor	Moderate I	Moderate I	Minor	Minor	Minor
Ct values	N1	20.51	28.52	24	23	28.19	22.51	17.95	19.82	25.65
	N2	21.14	28.84	23	25	28.86	23.25	18.47	19.97	26.06
Test sample*		S	S	N	N	S	S	S	S	S
Detailed information at the time of observation										
Days of illness		3	5	4	2	5	5	3	5	4
Fever (> 37.5°C)		+	+	+	+	-	+	+	+	-
Cough		+	-	+	+	-	+	+	+	-
Minimum recorded SpO_2_ during observation period (SpO_2_ < 93%)		+	-	+	-	-	-	+	-	-

*Test sample: S, saliva; N, nasopharyngeal swab specimen † Severity was assigned at hospital admission according to the national guideline and was not updated thereafter.

### Detection of SARS-CoV-2 RNA on environmental surfaces

Of 134 specimens, 66 (49.3%) were positive for SARS-CoV-2 RNA. The mean log_10_（copies） per sample was 3.88 (range, 1.43-8.48 log_10_). Textile items showed high detection frequencies—for example, masks and pajamas (100%) and pillowcases (88.9%)—and a bedside handrail yielded the highest RNA load (8.48 log_10_) (Table 2; Supplementary Table S1).

**Table 2:** Summary of severe acute respiratory syndrome coronavirus 2 RNA detection

Map No.	Object	RNA positives	Virus amount (RNA copies/sample, log_10_)			Residual infectivity	Distance from mouth (m)
			Mean	Max	Min		
A	Surgical mask	100% (9/9)	4.56	5.72	2.94	0% (0/9)	0
B	Pillowcase	88.9% (8/9)	4.21	5.92	1.91	0% (0/9)	0.1
C	Pajamas	100% (9/9)	4.35	7.00	2.33	22.2% (2/9)	0.2-0.5
D	Bed railing (overhead)	55.6% (5/9)	2.26	2.81	1.73	0% (0/9)	0.3
E	Smartphone screen	75.0% (6/8)	3.36	4.63	1.84	0% (0/9)	0.5*
F	Nurse call button	44.4% (4/9)	3.15	3.89	2.48	0% (0/9)	0.5
G	TV remote controller	55.6% (5/9)	3.60	4.62	2.06	0% (0/9)	0.7*
H	Bed railing (side)	55.6% (5/9)	3.75	8.48	2.34	11.1% (1/9)	0.8
I	TV stand	33.3% (3/9)	3.22	4.15	2.62	0% (0/9)	1.0
J	Light switch	33.3% (3/9)	3.19	3.73	2.38	0% (0/9)	1.1
K	Table	44.4% (4/9)	2.84	3.91	1.43	0% (0/9)	1.8*
L	Floor	11.1% (1/9)	3.21	3.21	3.21	0% (0/9)	2.4
M	Doorknob (shower room)	44.4% (4/9)	3.15	4.49	2.20	0% (0/9)	3.6
N	Doorknob (hospital room, inside)	11.1% (1/9)	4.53	4.53	4.53	0% (0/9)	4.4
O	Nurse’s scrub (upper back)	0% (0/9)	-	-	-	-	-

*Portable subject, † Smartphone screen samples were obtained from eight of the nine participants; one participant (ID #8) did not own a smartphone, and therefore no sample was collected (denominator = 8).

### Infectious virus and estimated infectious titers

Culture-based assessment across all samples identified infectious virus in four specimens (one bed rail and three sets of pajamas). RNA loads in these four specimens were 5.72-8.48 log_10_, approximately 70-40,000 times higher than the overall mean (3.88 log_10_). For culture-positive specimens, Ct values obtained by RT-PCR of culture supernatants from culture-positive specimens were used in the published regression, log_10_ (TCID_50_/mL) = −0.3152 × Ct + 9.988, to estimate infectious titers (TCID_50_-eq per sample)^10)^. Ct values ranged from 21.96 to 24.06, corresponding to estimated infectious titers of 5.7 × 10^2^ to 2.6 × 10^3^ TCID_50_-eq per sample (Supplementary Table S2).

### Distance dependence and contamination at distal sites

**Figure 2:**
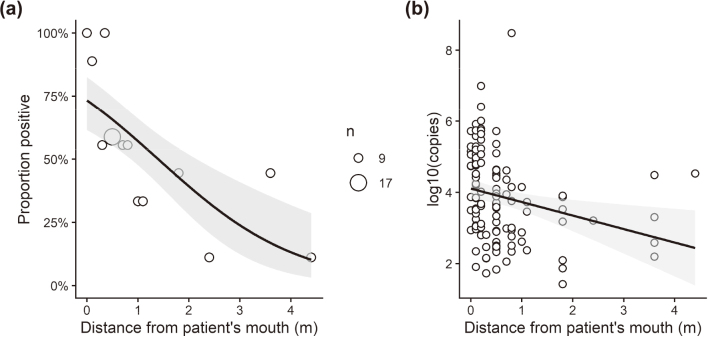
Distance-dependent decline in environmental SARS-CoV-2 RNA contamination (a) Proportion of RNA-positive samples as a function of distance from the patient, with fitted logistic regression. Circle size represents the number of samples within each distance category. (b) log_10_ RNA copies per sample by distance, with fitted linear regression. Greater distance from the patient was significantly associated with lower contamination: the odds ratio per 0.1 m was 0.93 (95% CI, 0.894-0.962; p < 0.001), and the linear slope was −0.379 log_10_ copies per meter (p = 0.0067). All analyses and figure generation were performed in R version 4.5.2.

**Figure 3:**
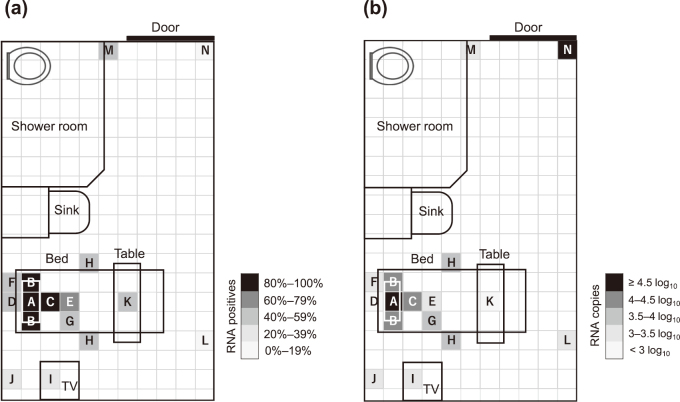
Viral RNA detection site mapping The hospital room was divided into 30 cm × 30 cm areas, and viral RNA detection sites were mapped. (a) Positivity rate, (b) viral RNA copy number. Locations A-N are the same as in Figure 1.

RNA detection exhibited clear distance dependence, decreasing progressively as the distance from the patient’s mouth increased. The odds ratio (OR) per 1-m increase in distance was 0.48 (95% CI, 0.33-0.68; *p* < 0.001), corresponding to an OR of 0.93 per 0.1-m increment in the original model. Consistently, RNA load decreased by -0.379 log_10_ copies per meter in linear regression (*p* = 0.0067), and Spearman’s ρ was -0.385 (*p* < 0.001) (Figure 2). Despite this distance-dependent decline, contamination remained detectable at distal high-touch surfaces, including the toilet doorknob (positivity 44.4%; mean 3.15 log_10_ copies per sample; 3.6-4.4 m from the mouth) and the room entrance doorknob (positivity 11.1%; mean 4.51 log_10_ copies) (Figure 3).

### Surface material effects

Compared with nonporous surfaces (positivity, 41.8%; 95% CI, 32.6-51.7), textiles demonstrated substantially higher positivity (96.3%; 95% CI, 81.7-99.3; Fisher’s exact test, *p* < 0.0001). RNA load was also higher on textiles (median, 4.45 log_10_ copies [IQR, 3.53-5.19]) than on nonporous surfaces (3.01 log_10_ copies [IQR, 2.48-3.88]; Wilcoxon test, *p* < 0.0001). Culture positivity did not differ significantly (nonporous, 2.1% vs textiles, 4.3%; Fisher’s exact test, *p* = 0.65). In a sensitivity model adjusting for distance, material type remained significantly associated with positivity (odds ratio for textiles vs nonporous, 20.6; 95% CI, 2.56-165.6; *p* = 0.0045) (Supplemental Table S3).

### Risk factors for severe conditions

In analyses stratified by severity classification (n = 9), older age demonstrated a clear inverse association with environmental contamination. With every additional decade of age, the odds of environmental detection declined by approximately 38% (OR, 0.618; 95% CI, 0.499-0.766; *p* < 0.001), and the mean RNA load decreased by 0.21 log_10_ copies (95% CI, −0.36 to −0.06; *p* = 0.029). Presence of cough was associated with higher detection (OR, 3.02; 95% CI, 1.40-6.54; *p* = 0.005). Other factors, including the Ct values for N1 and N2 at admission, were not significantly associated with either detection or RNA load. Regarding culture positivity, no clinical factor demonstrated a statistically significant association (e.g., age: OR, 0.51; 95% CI, 0.21-1.27; *p* = 0.15). Given the limited number of events, Firth’s logistic regression was applied for fever and cough, yielding ORs of 3.89 (95% CI, 0.21-7.68; *p* = 0.386) and 7.00 (95% CI, 0.42-1084.80; *p* = 0.192), respectively. (Supplementary Table S4).

## Discussion

In single-occupancy isolation rooms for patients with COVID-19, we frequently detected SARS- CoV-2 RNA shed by patients during the first 24 hours after admission and infectious virus was recovered from a subset of sample. Environmental contamination exhibited a clear distance-dependent gradient from the patient’s mouth.

We applied adhesive samplers immediately before patient entry and retrieved them 24 h later, after which samples underwent extraction and ultrafiltration prior to RNA detection and infectivity assays. The recovery of viruses from solution by ultrafiltration has previously been reported as approximately 56%^9)^. In our bench testing using influenza virus, recovery from the study samplers was 46% for adhesive seals and 31% for cloth patches. Considering the added extraction step, these values are within a reasonable range. Accordingly, while absolute quantities may be underestimated, relative comparisons across distance and material remain valid and interpretable.

In this study, the isolation rooms combined an outdoor air ventilation rate of 456 m^3^/h (≈12 ACH) for a room volume of 38 m^3^ with a HEPA-filter air cleaner providing an additional equivalent flow of 583 m^3^/h, constituting a high-ventilation environment, even compared with typical hospital rooms or negative-pressure rooms (6-12 ACH). During the COVID-19 pandemic, numerous instances of through- the-air transmission were reported^12-14)^, and ventilation and filtration have been emphasized as key measures to reduce inhalation risk^15, 16)^. Prior reports indicate that in high-ventilation rooms such as airborne infection isolation rooms, SARS-CoV-2 RNA is often undetectable in air samples, whereas environmental surfaces still yield RNA at rates of 5- 87%^17-19)^. Consistent with these observations, the positivity rate on environmental surfaces in our study was 49.3%, reaffirming that surface contamination can persist even under high-ventilation conditions.

With respect to the relationship between distance and viral contamination, both RNA positivity and RNA copy numbers decreased significantly with increasing distance from the patient’s mouth. This supports the premise that surfaces closer to the patient are subject to greater deposition of droplets and aerosols and suggests that distance may serve as a practical indicator for prioritizing areas in environmental cleaning.

The estimated infectious titers of the culture-positive specimens (5.7 × 10^2^-2.6 × 10^3^ TCID_50_-eq per sample) were broadly in line with previously reported ranges^6, 7)^. A human challenge trial conducted in 2021 showed that an intranasal dose of ~10 TCID_50_ infected about half of healthy adults, indicating that very small amounts can suffice under certain conditions^8, 20)^. However, in real-world fomite transmission the risk is expected to be markedly attenuated relative to direct deposition because two successive transfer steps are involved, surface to hands and hands to mucosa. Reported transfer efficiencies for fingertip-surface and surface-fingertip contacts, though variable by system and conditions, range from a few percent to multiples of ten percent^21, 22)^. Consequently, the dose that can reach mucosal surfaces after contact with a contaminated surface is likely to be orders of magnitude lower than the infectious titer present on the surface. Nevertheless, the titers estimated here could still exceed the minimal infectious dose even after a reduction on the order of one log. Furthermore, as people touch their faces a dozen to several dozen times per hour^23)^, fomite transmission may occur under conducive conditions.

By material type, both RNA detection positivity and RNA load were significantly higher on textiles than on non-porous surfaces. Infectious virus was detected on high-touch surfaces in close proximity to the patient’s mouth (one bed rail and three pajama samples) but not from patient-worn masks. Although prior laboratory studies have demonstrated rapid loss of infectious SARS-CoV-2 on cotton fabrics compared with non-porous surfaces^24, 25)^, these findings were generated under static, highly controlled conditions. In contrast, patient-care environments are dynamic, with substantial heterogeneity in exposure frequency, deposition load, and microenvironmental conditions. In our study, patient-worn masks—a warm, humid, and enclosed microenvironment—yielded no infectious virus, consistent with accelerated inactivation under high humidity and temperature^26)^. Conversely, textiles located immediately adjacent to the patient’s mouth (pajamas) were repeatedly and directly exposed to respiratory droplets and aerosols during talking, coughing, and breathing. Such high-frequency, high-load exposure may overcome the intrinsic rapid inactivation reported on cotton in laboratory settings, enabling recovery of infectious virus despite the porous nature of the material. These observations indicate that, in real-world clinical settings, environmental persistence is determined not solely by material properties but by the combined influence of exposure intensity, patient behavior, and local microenvironmental conditions.

In prespecified severity classification univariable analyses, increasing age was associated with lower levels of environmental contamination, whereas the presence of cough was positively associated with RNA detection. In contrast, respiratory failure, fever, and admission Ct values were not significant predictors of surface contamination. These findings should be interpreted with caution, given the small sample size and the use of univariable analyses, which allow for residual confounding and chance effects. Confirmation in larger prospective studies that incorporate behavioral metrics is warranted.

No RNA was detected on nurses’ scrub patches placed on the upper back. The study did not record time spent in patient rooms or frequency of patient contact; therefore, the association between caregiver exposure and contamination could not be assessed. Because caregiver presence and contact behaviors may strongly influence environmental contamination, future studies should incorporate time-activity recording and contact metrics.

The limitations of this study are as follows. To begin with, small, single-center study with a single 24-h sampling window, resulting in imprecise estimates. In addition, conversion from post-culture Ct values to TCID_50_ equivalents (TCID_50_-eq) depends on the reagents and viral strains used and therefore does not directly represent the infectious titer present in the original environmental samples^10, 27)^. Furthermore, the study was conducted in 2021 and focused on the then-predominant Delta variant, which differs from currently circulating Omicron- lineage variants. Because surface persistence may differ by variant^28)^ and we did not perform strain- specific analyses, extrapolation should consider temporal context and variant characteristics. Although near-mouth surfaces generally exhibited higher contamination, two cloth samples (#1 and #2) demonstrated comparatively low detection. This pattern may not necessarily reflect reduced viral burden but could instead result from heterogeneity in recovery efficiency on textile materials or local microenvironmental effects (e.g., airflow turbulence, drying kinetics). Accordingly, these results should be interpreted with caution, and potential sampling or recovery bias cannot be excluded.

## Conclusion

Environmental sampling in high-ventilation single rooms revealed that SARS-CoV-2 RNA was detected predominantly on high-touch surfaces in close proximity to patients, including textile materials, and infectious virus occasionally persisted in the environment. Based on estimated infectious titers, even after accounting for attenuation across transfer steps from environmental surfaces to the mucosa, implying that fomite transmission remains plausible, particularly when high-touch surfaces near patients are not adequately cleaned or disinfected. While ventilation effectively mitigates through-the- air transmission, high-touch surfaces near patients require targeted cleaning and disinfection and consistent hand hygiene. Future studies should further clarify how material type and microenvironmental factors (e.g., temperature and humidity) affect viral stability, and incorporate behavioral logging in prospective observational studies to improve risk assessment and inform more effective strategies to mitigate fomite-mediated transmission.

## Author contributions

Conceptualization: AH, KM, TM, SH.

Data curation: AH, MS, MH.

Formal analysis: AH.

Investigation: AH, YO, SK.

Methodology: AH, MO, TN, YT, TM, NO, SH.

Project administration: NO, SH.

Supervision: SH.

Validation: AH, MO, TM, SH.

Writing—Original draft preparation: AH, TM.

Writing—Review & editing: MO, MH, YO, SK, MS, TN, YT, KM, TM, NO, SH.

All authors read and approved the final manuscript.

## Conflicts of interest statement

AH, YO, SK, KM, TM, NO are employees of Kao Corporation, which provided technical assistance, experimental equipment, and support for data analysis and interpretation. The authors declare no conflicts of interest.

## Supplementary Material

**Supplementary Table S1:** Severe acute respiratory syndrome coronavirus 2 RNA copy number (log_10_/cps*) in samples

Map No.	Object		#1	#2	#3	#4	#5	#6	#7	#8	#9
A	Surgical mask		3.50	5.16	5.06	5.72	2.94	5.29	4.74	3.87	4.75
B	Pillowcase	Upper left	ND	3.25	5.22	4.76	1.91	4.97	3.07	3.55	4.52
		Lower left	ND	ND	5.58	5.74	2.35	3.84	5.20	3.05	4.79
		Upper right	ND	ND	5.92	5.07	NR	4.21	3.84	3.41	3.52
		Lower right	ND	ND	5.29	4.91	2.97	4.24	4.50	4.42	3.85
C	Pajamas	Chest area_left	ND	3.62	6.41	6.01	2.80	7.00	3.68	5.07	4.83
		Chest area_right	ND	3.02	5.48	5.79	3.12	5.23	4.02	5.74	5.64
		Sleeve_left	2.61	3.57	5.10	5.72	2.92	4.64	2.33	3.71	3.97
		Sleeve_right	3.64	NR	4.52	5.36	2.44	4.47	2.90	4.10	3.97
D	Bed railing (overhead)		ND	ND	2.81	2.15	ND	1.73	ND	2.16	2.47
E	Smartphone screen		ND	2.51	4.63	4.41	ND	3.59	1.84	NR	3.17
F	Nurse call button		ND	ND	3.34	2.90	ND	3.89	2.48	ND	ND
G	TV remote controller		ND	ND	2.99	4.62	ND	4.36	ND	2.06	3.95
H	Bed railing (side)	Left	ND	ND	4.15	2.95	2.34	2.77	ND	ND	ND
		Right	ND	ND	8.48	3.76	ND	3.01	ND	ND	2.51
I	TV stand		ND	ND	2.62	2.88	ND	4.15	ND	ND	ND
J	Light switch		ND	ND	3.46	3.73	ND	ND	2.38	ND	ND
K	Table	Left	ND	ND	3.87	3.18	ND	2.10	ND	ND	ND
		Right	ND	ND	3.91	3.53	ND	1.87	1.43	ND	ND
L	Floor		ND	ND	3.21	ND	ND	ND	ND	ND	ND
M	Doorknob (shower room)		ND	ND	4.49	2.59	ND	3.31	2.20	ND	ND
N	Doorknob (hospital room, inside)		ND	ND	4.53	ND	ND	ND	ND	ND	ND
O	Nurse’s scrub (upper back)		ND	ND	ND	ND	ND	ND	ND	ND	ND

ND, not detected, NR, not recovered Infections samples are denoted by bold and underlined numbers. *Cps, copies per sample (36 cm^2^) In this study, “NR” resulted either from issues in sample collection (subjects #2, and #5) or from the participant not carrying a smartphone (subject #8).

**Supplementary Table S2:** Estimated infectious titer per sample based on post-culture Ct valves

ID	Object	Ct values in post culture	Estimated infectious titer (TCID_50_-eq/sample)
#3	Bed railing	23.87	655.2
#4	Pajama	24.06	570.8
#4	pajama	23.76	709.6
#6	pajama	21.96	2620.5

**Supplementary Table S3:** Outcomes stratified by material

Material	RNA positivity (%) with 95% CI	RNA copies median [IQR]	Culture positivity (%) with 95% CI
Non-porous	41.8 (41/98) [32.6-51.7]	3.01 [2.48-3.88]	2.1 (1/47) [0.4-11.1]
Textiles	96.3 (26/27) [81.7-99.3]	4.45 [3.53-5.19]	4.3 (3/70) [1.5-11.9]
*p*-value	< 0.0001 (Fisher’s exact)	< 0.0001 (Wilcoxon)	0.648 (Fisher’s exact)

**Supplementary Table S4:** Associations of patient-level factors with environmental outcomes

Outcome	Predictor	Effect (95% CI)	*p*-value
RNA detection (positivity)	Age band	OR = 0.618 (0.499 - 0.766)	< 0.001
	N1 Ct (per 1 Ct)	OR = 0.956 (0.862 - 1.059)	0.387
	N2 Ct (per 1Ct)	OR = 0.951(0.859 - 1.053)	0.334
	Days of illness onset	OR = 0.756 (0.537 - 1.065)	0.110
	Fever (yes vs no)	OR = 2.111 (0.894 - 4.982)	0.088
	Cough (yes vs no)	OR = 3.019 (1.395 - 6.535)	0.005
	Respiratory failure (yes vs no)	OR = 1.240 (0.587 - 2.619)	0.572
RNA load (mean log_10_ copies)	Age band	β = -0.210 (-0.361 - -0.060)	0.029
	N1 Ct (per 1 Ct)	β = -0.012 (-0.134 - 0.110)	0.851
	N2 Ct (per 1Ct)	β = -0.018(-0.163 - 0.127)	0.778
	Days of illness onset	β = -0.118 (-0.507 - 0.271)	0.571
	Fever (yes vs no)	β = 0.469 (-0.481 - 1.419)	0.366
	Cough (yes vs no)	β = 0.446 (-0.383 - 1.275)	0.327
	Respiratory failure (yes vs no)	β = -0.009 (-0.901 - 0.883)	0.984
Infectious virus (culture)	Age band	OR = 0.511 (0.206 - 1.266)	0.147
	N1 Ct (per 1 Ct)	OR = 0.977 (0.652 - 1.464)	0.912
	N2 Ct (per 1Ct)	OR = 0.988(0.663 - 1.474)	0.955
	Days of illness onset	OR = 0.639 (0.169 - 2.421)	0.510
	Fever (yes vs no)	OR = 3.889 (0.211 - 607.678)	0.386
	Cough (yes vs no)	OR = 7.000 (0.415 - 1084.804)	0.192
	Respiratory failure (yes vs no)	OR = 1.000 (0.053 - 18.916)	1.000

Effect estimates are odds ratios for RNA detection and infectious virus, and regression coefficients (β) for RNA load.
